# Introduction and behavioral validation of the climate change distress and impairment scale

**DOI:** 10.1038/s41598-023-37573-4

**Published:** 2023-07-12

**Authors:** Johanna Hepp, Sina A. Klein, Luisa K. Horsten, Jana Urbild, Sean P. Lane

**Affiliations:** 1 Department of Psychosomatic Medicine and Psychotherapy, Central Institute of Mental Health, Medical Faculty Mannheim, Heidelberg University, Mannheim, Germany; 2 Systems Neuroscience in Psychiatry, Central Institute of Mental Health, Medical Faculty Mannheim, Heidelberg University, Mannheim, Germany; 3 Experimental Psychology and Personality, RPTU Kaiserslautern-Landau, Landau, Germany; 4 Department of Psychological Sciences, University of Missouri, Columbia, MO USA

**Keywords:** Psychology, Climate change

## Abstract

Governmental agencies and the medical and psychological professions are calling for a greater focus on the negative mental health effects of climate change (CC). As a first step, the field needs measures to distinguish affective/emotional distress due to CC from impairment that requires further scientific and diagnostic attention and that may require treatment in the future. To this end, we constructed the *climate change distress and impairment scale*, which distinguishes CC distress (spanning anger, anxiety, and sadness) from impairment. In four studies (*N* = 1699), we developed and validated English and German versions of the scale. Across samples, spanning 2021–2022, CC distress was at least moderate, while we observed general moderate to high levels of distress and low to moderate levels of impairment. In three English-speaking samples, younger individuals and women were most affected by CC distress, whereas this was not the case in a German-speaking sample, suggesting sociopolitical influencing factors. We demonstrate convergent validity with previous measures and discriminant validity for general negative affectivity and depressive and generalized anxiety disorder symptoms, which underlines that CC distress is not in itself pathological. Employing a fully incentivized social dilemma paradigm, we demonstrate that CC distress and (to a lesser degree) CC impairment predict pro-environmental behavior, underscoring them as possible drivers, and targets, of climate-change mitigation efforts.

## Introduction

The effects of climate change, including the warming of the atmosphere, the loss of biodiversity, and increases in natural disasters, are the central crisis humanity faces in the current century^1,2^. With the effects of climate change unequivocally progressing, recent years have seen increased public^3^ and scientific^4^ interest in the negative consequences climate change has for people’s mental health, and there is now substantial evidence linking the effects of climate change to increased rates of psychopathology^5^. Importantly, climate change is also associated with negative psychological outcomes below the threshold of psychopathology. Most prominently, these include negative affect in response to climate change^6,7^. Specific types of negative affect in response to climate change that have been defined and investigated include *eco-anxiety or climate anxiety* (anxiety about the consequences and progression of climate change and a sense of threat^4^), *ecological grief* (grief as a response to environmental degradation and loss of species or beloved environments^8^), and *solastalgia* (distress and sense of loss due to change to a home environment^9^). Recent work has further coined the terms *eco-depression* and *eco-anger,* though these constructs currently still lack a clear definition^10^.

Although negative affective reactions to climate change can be associated with mental health issues^11^, they are not pathological in and of themselves. Instead, they are considered a natural reaction to an existential threat^9^ and may even be fundamental drivers of adaptive behavior such as pro-environmental action. Indeed, cross-sectional surveys have repeatedly linked climate anxiety to pro-environmental behavior^12,13^, and there is growing evidence that anger and sadness in response to climate change, as well as feelings of guilt, may also promote pro-environmental behavior^10,14,15^. In order not to pathologize negative affect in response to climate change, it will be crucial to distinguish it from functional impairment experienced in response to climate change. For instance, if an individual’s ability to work or attend school, maintain social relationships, or uphold a daily structure is impaired because of their preoccupation with climate change, they may require treatment.

Past work has made important progress in measuring affective reactions to climate change^7,10,16,17^ but no measure has yet been published that distinguishes the affective reaction from an impairment component. The only measure that, to date, addresses impairment is the *climate anxiety scale*^18^. The scale comprises subscales for cognitive-affective impairment, functional impairment, climate change exposure, and behavioral (pro-environmental) engagement. The two impairment subscales together form the climate anxiety scale. Thus, the anxiety and the impairment component are not separated in this inventory; but, critically, it highlights the central role of impairment. During the scale development, the authors also asked participants to indicate to what degree they felt sad, scared, alone, angry, pessimistic, guilty, helpless, hopeless, isolated, frustrated, or resigned in response to climate change. The cognitive-affective impairment subscale correlated strongly with the mean of these items, whereas the other three subscales correlated moderately with it. While this indicates the scale’s relevance for negative affect in response to climate change, the breadth of these negative affect items is not reflected in the final scale content.

### Present studies

The aims of the present studies were threefold. *First*, we aimed to develop a scale that covers different types of negative affect in response to climate change. We included anxiety, sadness, anger and guilt to, (i) reflect previous work showing these are all affects relevant to climate change, including work on eco-/ climate change anxiety, eco-depression, solastalgia, ecological grief, and eco-anger, (ii) cover affects that were linked to pro-environmental behavior in previous work^10,12,14,15^, and (iii) reflect common sources of psychopathology in order to critically test whether negative affect in response to climate change is distinct from psychopathology. We included anger as the central affect relevant to externalizing pathology and anxiety, sadness, and guilt, as emotions central to internalizing pathology^19^.

Henceforth, we will refer to the group of these four negative affects as *climate change distress* (CCD). The APA dictionary of psychology defines distress as “the negative stress response, often involving negative affect and physiological reactivity: a type of stress that results from being overwhelmed by demands, losses, or perceived threats”^20^. Thus, the term distress well reflects negative affect in response to the specific, existential stressor that climate change represents. In addition to distress, we aimed to develop items to measure *climate change impairment* (CCI) that cover general, social, and work/school related impairment. We tested the initial item pool of the resulting *Climate Change Distress and Impairment Scale* (CC-DIS) in Study 1, validated the factor solution in Study 2, and established structural, convergent, and discriminant validity in Study 3.

*Second*, we aimed to investigate the criterion validity of the CC-DIS by testing whether it predicts pro-environmental behavior (PEB). PEB is defined as behavior that consciously aims at avoiding or minimizing one's negative impact on the natural world^21,22^. Recent work^10,12,14,15^ identified affective responses towards climate change as one of the key drivers of PEB and support for climate change mitigation policy. Therefore, we hypothesized that CCD and CCI would be associated with increased levels of PEB. We tested this in Study 4, where we measured PEB in an incentivized social dilemma.

*Third,* we aimed to assess the prevalence of CCD and CCI in different demographic groups. We hypothesized that CCD and CCI would show a negative association with age, such that younger individuals are more affected. This follows from previous work showing that younger individuals (aged 18–36) repeatedly reported increased levels of negative emotions in reaction to climate change^7,18,23,24^. Moreover, in a recent sample of 10,000 young persons from ten socio-culturally diverse countries across the globe, more than 45% of participants (aged 16–25) stated that their feelings about climate change negatively affected their daily lives, suggesting increased levels of CCI in this age cohort^25^. In addition to age, we assessed effects of gender, aiming to replicate previous findings of increased rates of negative climate change-associated emotions in women^7,18^. As women appear proportionately more vulnerable to the consequences of climate change^26^, we expected higher levels of CCI than in men.

## Study 1: item generation and selection

JH and SAK developed the initial item pool of 84 items covering *anxiety* about the consequences of climate change, *sadness* about the losses already incurred by climate change, *anger* about the climate-related actions of other individuals or entities, and *guilt* about one’s own role in climate change. The inclusion of these four negative affects reflects the breadth of emotions relevant to climate change^10,27^, follows established emotion models^28^, and reflects common sources of psychopathology^29^. Sixteen items each covered anger, sadness, anxiety, and guilt with half the number of items reverse-coded. Twenty items covered general impairment, social impairment, and work/school related impairment (again, half were reverse-coded). Next, we sought feedback on the cultural relevance of the item content and item comprehensibility from English native speakers in Australia, India, Ireland, South Africa, and the USA and adjusted the wording of several items. For the full item list, see supplemental Table S1a–e (all supplemental materials available at https://osf.io/eprdw/).

### Method

#### Procedure

Ethics approval for Studies 1–3 was granted by the medical ethics committee II at Heidelberg University (ID 2021-543). All research was performed in accordance with the regulations specified in the ethics approval and in accordance with the Declaration of Helsinki. In all three studies, participants provided informed consent prior to participation and were guaranteed complete anonymity. Studies were conducted online via the panel provider *prolific.co*. For Study 1, we recruited 403 participants for a study on “negative emotions and climate change” in May 2021. After providing consent, participants answered demographic questions and completed the CCD and CCI items. Items were answered on a 5 point Likert-type scale (1-strongly disagree, 2-disagree, 3-neutral [neither agree nor disagree], 4-agree, 5-strongly agree). Mean completion time was 10.14 min and participants were paid £1.25, which equals the £7.50/hr wage recommended by Prolific^©^ as of May 2021.

#### Participants

Inclusion criteria were being 18 years of age or older and fluent in English. The sample was recruited internationally. Following data collection, we excluded 19 total participants (4.7%) from the analyses: one due to a low completion time (on average less than 2 s per CC-DIS item, using the average time spent on the survey pages with the CC-DIS items), and 18 because they indicated they did not believe in anthropogenic climate change. Participants who do not believe in anthropogenic climate change were excluded for the initial scale development because we expected the majority of items not to apply to them, or for them to experience distress for reasons other than those covered by the scale (e.g., as a result of mitigation/education efforts they do not support). This group should be studied further, but was beyond the scope of the initial scale development. The final sample consisted of *N* = 384 participants. Detailed demographic data (gender, age, race, language, education, employment, income, pathology, and country of residence) is presented in Table S2. Participants were aged 18 to 65 years (*M* = 26.41, *SD* = 8.68), with the majority of participants identifying as male (58.3%), and white (88.5%). All participants were either native speakers (16.7%) or fluent (83.3%) in English. The large majority of participants (82.3%) were from continental Europe.

#### Data analysis

Analyses for all studies were conducted in R. Data and code are available at https://osf.io/eprdw/. Following guidelines for scale construction^30^, in the first step of item selection we excluded only the worst performing items to moderately decrease the item pool. First, we recoded reverse-coded items so that higher scores reflected greater distress or impairment. We then excluded items that correlated below < 0.20 with other items of their designated subscale. If several items correlated < 0.20, we excluded items successively, starting with the item with the most inter-item-correlations < 0.20. The guilt items performed poorly overall, with many of them showing null or even negative correlations with other items. In addition to inter-item correlations, we took into account item distributions and scale content (retaining some items to maintain breadth of the construct and excluding others that closely overlapped with other items or did not fit the construct as well). See Table S3 and R script for a detailed description of exclusion decisions for each item, Tables S4, S5, S6, S7 and S8 for correlation matrices and the Supplemental Figure folder on *osf* for boxplots. After this initial step of item selection, we retained 15 anger, 11 sadness, 12 anxiety, 4 guilt, and 15 impairment items.

Using the remaining item pool, we performed an exploratory factor analysis (EFA) using the *psych* package with maximum likelihood extraction and Varimax rotation. Based on our a-priori reasoning that items reflected the four negative affects anger, sadness, anxiety, and guilt, as well as an impairment factor, we initially extracted five factors, aiming to investigate whether the four affects would form distinct subscales.

### Results

The factor-loading matrix for the five-factor solution is presented in Table S9a–c. Overall, the five-factor solution was not easily interpretable and did not reflect the hypothesized factor structure with one factor for each negative affect and one for impairment. The screeplot (see Supplemental Figures) suggested a two-factor solution as a more appropriate representation of the factor structure. We therefore conducted another EFA, extracting two factors (see Table S10a–c). The factor solution showed that all except five negative affect items loaded > 0.35 on the first factor that accounted for 23% of variance in item ratings. All impairment items showed factor loadings > 0.42 on the second factor that accounted for 14% of variance. Three sadness items and one guilt item had cross-loadings on Factor 2. Their content reflected lack of control or depression-like feelings (e.g., “Climate change makes me feel hopeless.”), which is similar to findings by Searle and Gow^7^ who, for their inventory, found a hopelessness factor and an anxiety factor. A retroactive two factor confirmatory factor analysis (CFA) including only the final items corresponding to distress (anger, anxiety, sadness) and impairment determined in Study 2 indicated adequate overall model fit (CFI = 0.916, TLI = 0.903, SRMR = 0.066, RMSEA = 0.061) and excellent factor-specific reliability, replicability, and determinacy (Ω_Distress_ = 0.841, Ω_Impairment_ = 0.860, *H*_Distress_ = 0.887, *H*_Impairment_ = 0.880, *FD*_Distress_ = 0.942, *FD*_Impairment_ = 0.938)^31,32^.

## Study 2: structural validation of the CC-DIS

In Study 1, we observed that the structure underlying our items was best represented by a two-factor solution reflecting CCD (across all four affects) and CCI, rather than four specific affect scales. Additionally, we observed that the guilt items underperformed psychometrically. We reasoned that this may have resulted from the reverse-coded guilt items not appropriately reflecting low or absent guilt, but rather a state of contentment with one’s own actions regarding climate change. Some items could also have performed poorly because they more strongly reflected attitudes about CC rather than emotions resulting from it (e.g. “I feel that I am not doing enough to stop climate change”). Lastly, climate change-associated guilt itself may heavily depend on the individual’s appraisal of whether and how much they can contribute to mitigating climate change. If an individual appraises their own behavioral options as negligible, they may experience minimal guilt even though they are otherwise emotionally affected by climate change. Nonetheless, before discarding the guilt construct entirely, we decided to develop a set of 11 new guilt items to add to the item pool administered in Study 2 (see Table S11a–e for item list).

### Method

#### Procedure

Study advertisement, payment scheme, and procedure were identical to Study 1. Data were collected in September of 2021. Mean completion time was 7.04 min and participants were again paid £1.25. As participants were faster than the expected 10 min (*M* = 7.04, *SD* = 2.12); their payoff equaled a £10.79/hr wage. A filter implemented on *prolific.co* ensured that participants who completed Study 1 were not able to participate in Study 2. Unlike Study 1, we only allowed native English speakers in Studies 2 and 3.

#### Participants

We recruited 450 native English speakers, who all indicated they believed in anthropogenic climate change. After excluding three participants due to a low completion time, the final sample included *N* = 447 participants. Participants were aged between 18 and 75 years (*M* = 33.64, *SD* = 12.62), and a majority identified as female (52.8%) and white (78.3%). Most participants (43.4%) were from the USA, followed by the UK (32.0%) and Canada (12.8%). For detailed demographic data, see Table S2.

#### Data analysis

First, we conducted an EFA to determine whether, with the new guilt items, the factor structure approximated five factors or whether the two factor solution would again better represent the data. We did this before inspecting inter-item correlations to determine whether we should consider inter-item correlations within the four specific negative affects or across them. The Varimax rotated factor-loading matrix for the five-factor solution is presented in Table S12a–d. As in Study 1, this factor solution did not well-reflect four affect subscales. We thus repeated the EFA, extracting two factors using maximum likelihood, and the Varimax rotated factors again indicated a distress and an impairment factor (see Table S13a–d for the factor loading matrix and online supplement for the screeplot). The factor solution indicated that inspection of inter-item correlations should be conducted within the group of distress and impairment items (not considering the four different negative affects). Mirroring the procedure in Study 1, we excluded items with inter-item correlations < 0.20, as well as poorly distributed items with restricted variance or pronounced skewness. During this step, we took into account item content in order to retain breadth of the construct. See Table S3 for details on item exclusion based on these indices, Tables S14 and S15 for correlation matrices and online supplement for boxplots. In this step, all guilt items were removed. The new set of guilt items also showed very low, and in many cases negative, correlations with the other affect items. Hence, the initial goal of covering CC-related guilt was set aside, as the guilt items performed poorly psychometrically in both samples.

Using the 42 items that remained following the initial selection process, we performed an EFA, extracting two factors using maximum likelihood and performing Varimax rotation (see Table S16a,b for the factor loading matrix). Following guidelines for scale construction^30,33^ we excluded items with main loadings < 0.50 or cross-loadings > 0.35. Following this, 36 items remained. Since we aimed for a scale with an equal coverage of anger, anxiety, and sadness items and an overall length that allows inclusion in large-scale surveys (which typically have very limited space), we decided to select the best 5 items per affect to cover the breadth of the distress construct. Additionally, we selected the 8 best performing impairment items. Thus, we retained 23 items, including five anger, sadness, and anxiety items each, and eight impairment items (6 general, 1 social, 1 work-related). We took care to retain not only positively, but also reverse-coded items, which resulted in the final scale comprising nine reverse-coded items. Table 1 shows the final item set.

**Table 1:** Final item set in English.

Item	Item text
	Distress
1	I feel angry when I see how little is done to combat climate change.
2	When I think about climate change, I worry about the future.
3	I am not sad about climate change. (r)
4	I am enraged that we have missed many chances to stop climate change.
5	I do not fear for my future on this planet. (r)
6	News about climate change makes me feel depressed.
7	I am not mad when others damage the climate. (r)
8	The uncertainty about how climate change will progress scares me.
9	I feel sad that climate change is causing people and animals to suffer.
10	I do not get upset when others ignore climate change. (r)
11	I am scared that people will lose their homes because of climate change.
12	I feel sad that some parts of the environment will not recover from the effects of climate change.
13	I am not angry that some countries have missed their climate protection goals. (r)
14	The impact that climate change has had on the planet saddens me.
15	I feel carefree when I think about climate change. (r)

	Impairment
16	Climate change drains all my energy.
17	My thoughts and feelings about climate change do not affect how well I sleep. (r)
18	When I think about climate change, I get a headache or stomachache.
19	Because of climate change, I am overwhelmed by everyday activities.
20	My thoughts and feelings about climate change do not negatively impact my everyday life. (r)
21	I have no trouble mentally tuning out climate change. (r)
22	Constant discussions about climate change are affecting my relationships.
23	When I think about climate change, I cannot bring myself to work/study.

*Note.* For each item, participants endorsed their level of agreement ranging from strongly disagree (1), disagree, neutral (neither agree nor disagree), agree, to strongly agree (5).

With the final item set, we performed a CFA, specifying a latent factor for distress (onto which the anger, sadness, and anxiety items should load), an impairment factor (onto which all impairment items should load), and a method factor onto which all reverse-coded items were specified to load^34^. We fixed the variances of the latent factors to 1 and estimated covariances between the latent factors.

### Results

Variances, covariances, and path coefficients for the CFA on the final item set are presented in Table S17a–e. Fit indices indicated adequate model fit (CFI = 0.904, TLI = 0.888, SRMR = 0.069, RMSEA = 0.072) and excellent factor-specific reliability, replicability, and determinacy (Ω_Distress_ = 0.914, Ω_Impairment_ = 0.888, *H*_Distress_ = 0.923, *H*_Impairment_ = 0.909, *FD*_Distress_ = 0.961, *FD*_Impairment_ = 0.953). All distress items loaded > 0.425 onto the distress factor. All impairment items loaded > 0.512 onto the impairment factor. All reverse-coded items loaded > 0.213 on the method factor. The distress and the impairment factor were moderately positively correlated (*r* = 0.287, *p* < 0.001). The method factor did not significantly correlate with either the distress factor (*r* = 0.085, *p* = 0.219) or the impairment factor (*r* = − 0.124, *p* = 0.117), substantiating its inclusion to primarily account for response bias.

## Study 3: convergent and discriminant validity

In Study 3, we aimed to establish convergent and discriminant validity of the CC distress and CC impairment subscales. For this purpose, we asked participants to respond to the CCD and CCI items and other self-report scales that measure CCD- and CCI-like constructs for convergent validity, and measures for similar but theoretically distinct constructs surrounding negative affectivity and nature relatedness.

### Method

#### Procedure

Recruitment strategy, study advertisement, payment scheme, and filters mirrored those of Studies 1 and 2. Data were collected in September of 2021. Participants first completed demographic information and the CCD and CCI items, and then completed measures to assess convergent and discriminant validity that were presented in random order. Mean completion time was 20.02 min (*SD* = 7.13) and participants were paid £7.50/hr.

#### Participants

We aimed to include a more racially diverse sample than in Studies 1 and 2, and initially recruited 401 native English speakers, of which we excluded seven due to a low completion time and 20 because they indicated they did not believe in anthropogenic climate change (6.7% overall). The final sample included *N* = 374 participants aged between 18 and 59 years (*M* = 30.71, *SD* = 9.18). A majority of participants identified as female (50.3%) and white (49.5%). Most participants (42.5%) were from the UK, followed by the USA (34.2%) and Canada (9.4%). For demographic details, see Table S2.

#### Measures

A detailed description of all measures used to assess convergent and discriminant validity is provided in the online supplement. To establish *convergent validity* of CCD, we included the seven items on distress about the consequences of climate change by Hornsey and colleagues^16^. Additionally, we included the 22-item climate anxiety scale^18^, expecting CCI to show convergent validity with the cognitive-emotional impairment and functional impairment subscales from this measure. We expected the correlations between CCD and the items by Hornsey and colleagues^16^, as well as the correlations between CCI and the impairment subscales to be higher than the correlations observed for measures of discriminant validity.

To examine *discriminant validity in the clinical domain,* we aimed to establish whether CCD and CCI are distinct, climate change-specific constructs rather than a by-product of a general negative appraisal of the world, such as occurring during major depression or generalized anxiety. To this end, we assessed symptoms of major depression with the 21-item Beck-Depression-Inventory II (BDI-II^35^) and included a screener for symptoms of generalized anxiety disorder, the GAD-7^36^. Beyond this, we aimed to establish that CCI measures a construct distinct from a low general quality of life as assessed by the 8-item EUROHIS-QOL^37^. Additionally, we deemed it important to demonstrate that CCD is distinct from general negative affect (and not just a facet of a generally negative affective landscape), and therefore measured positive and negative affect with the 60-item PANAS-X^38^, as well as neuroticism with the 12 item neuroticism subscale of the NEO-FFI^39^.

Lastly, we aimed to establish *discriminant validity in the environmental domain* and show that CCD and CCI are distinct from nature relatedness, assessed with the Nature Relatedness Scale^40^, and environmental attitudes, assessed with the Revised New Environmental Paradigm Scale^41^.

### Results

Boxplots for the final item set are presented in the online supplement. Correlation tables are presented in Tables S18 and S19. We repeated the CFA with the same specifications as in Study 2 (latent distress, impairment, and method factors). Variances, covariances, and path coefficients are presented in Table S20a–d. Fit indices indicated good fit (CFI = 0.939, TLI = 0.930, RMSEA = 0.057, SRMR = 0.074) and excellent factor-specific replicability, and determinacy (*H*_Distress_ = 0.927, *H*_Impairment_ = 0.900, *FD*_Distress_ = 0.963, *FD*_Impairment_ = 0.949). As in Study 2, the distress and impairment factors were mildly positively correlated (*r* = 0.192, *p* = 0.001), whereas the distress and the method factor were not significantly correlated (*r* = − 0.127, *p* = 0.072) and neither were the impairment and the method factor (*r* = − 0.007, *p* = 0.913). All distress items loaded > 0.360 onto the latent distress factor, all impairment items loaded > 0.458 onto the impairment factor, and all reverse-coded items loaded > 0.475 onto the method factor. Internal consistency for the distress scale was excellent with α = 0.92, Ω = 0.92. Internal consistency for the impairment scale was high with α = 0.89, Ω = 0.88.

**Figure 1:**
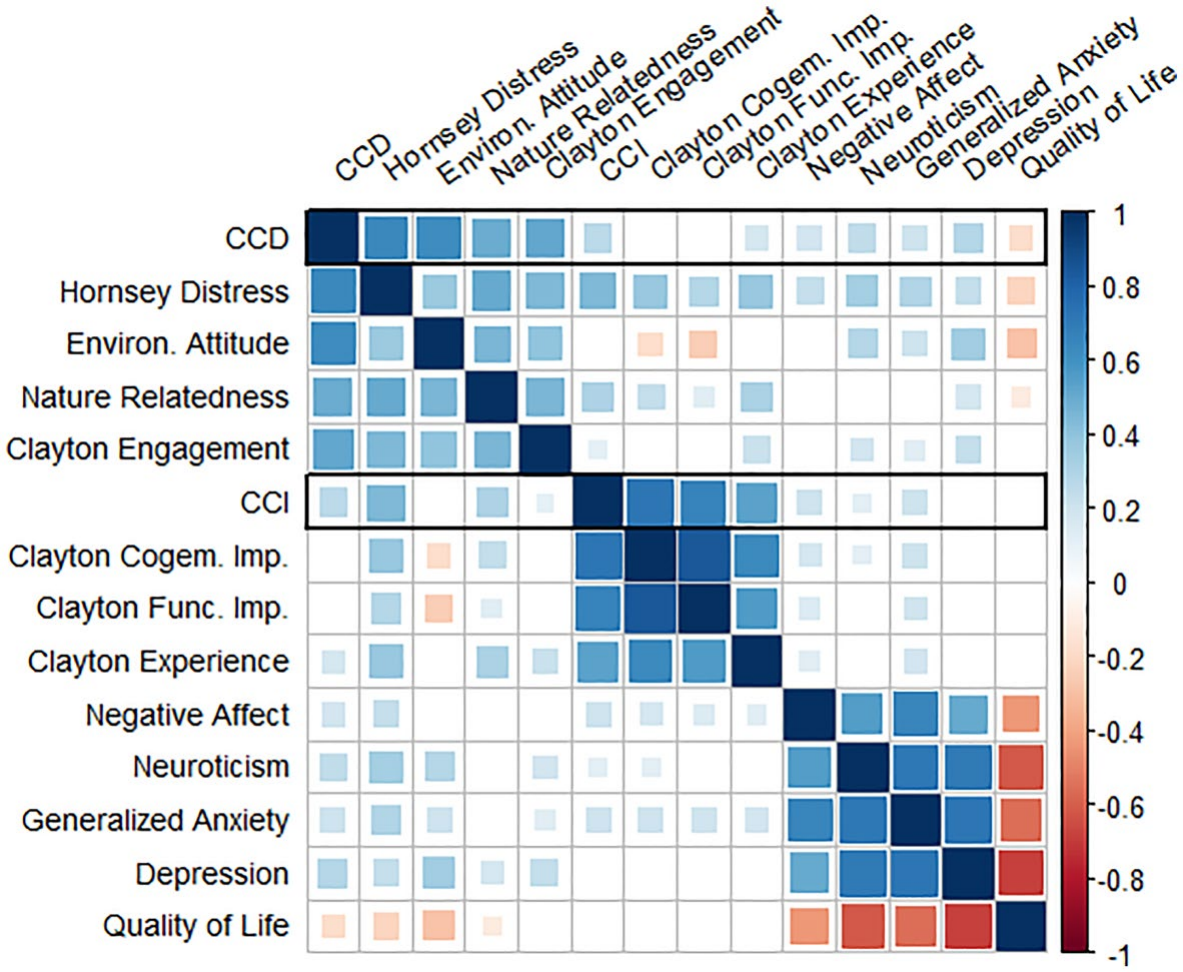
Correlation plot for CCD, CCI and constructs included for convergent and discriminant validity (size and color indicate magnitude of the pairwise correlation).

Next, we tested convergent and discriminant validity (see Table 2 and Fig. 1). As hypothesized, CCD showed the highest correlation with distress about the consequences of climate change^16^ at *r* = 0.64. CCI showed the highest correlations with cognitive-emotional impairment (*r* = 0.72) and functional impairment (*r* = 0.66), as measured with the climate change anxiety scale^18^. Thus, *convergent validity* for CCD and CCI was supported. *Discriminant validity* for CCD and CCI was established in the clinical domain, as depression, GAD, negative affect, neuroticism, and quality of life all correlated < 0.30 with CCD and < 0.12 with CCI. CCI also showed a near null correlation with environmental attitudes (*r* = − 0.09) and a moderate correlation with nature relatedness (*r* = 0.30), suggesting discriminant validity in the environmental domain. For CCD, the picture was less clear, as it correlated strongly with environmental attitudes (*r* = 0.62) and moderately with nature relatedness (*r* = 0.49).

**Table 2:** Study 3 correlations between CCD, CCI, and constructs included for convergent and discriminant validity.

	CCD	Hornsey distress	Environ. Attitude	Nature Related	Engage	CCI	Cogem. Impair	Func. Impair	Exper	NA	N	GAD	DD
CCD	1.00												
Hornsey distress	0.64	1.00											
Environ. Attitude	0.62	0.36	1.00										
Nature Related	0.49	0.50	0.45	1.00									
Engage	0.51	0.44	0.39	0.45	1.00								
CCI	0.26	0.44	− 0.09	0.30	0.10	1.00							
Cogem. Impair	0.10	0.37	− 0.18	0.23	0.08	0.72	1.00						
Func. Impair	− 0.05	0.28	− 0.25	0.12	− 0.02	0.66	0.84	1.00					
Exper	0.17	0.37	0.01	0.31	0.21	0.53	0.63	0.57	1.00				
NA	0.18	0.23	0.09	0.04	0.05	0.20	0.17	0.15	0.12	1.00			
N	0.24	0.33	0.28	0.10	0.18	0.11	0.11	0.09	0.09	0.55	1.00		
GAD	0.20	0.29	0.20	0.09	0.13	0.20	0.20	0.19	0.18	0.65	0.71	1.00	
DD	0.28	0.23	0.34	0.17	0.23	0.04	0.02	0.02	0.05	0.50	0.70	0.72	1.00
Quality of life	− 0.18	− 0.22	− 0.29	− 0.11	− 0.01	− 0.04	0.01	− 0.05	− 0.04	− 0.44	− 0.62	− 0.55	− 0.69

*CCD* climate change distress, *Environ. Attitude* environmental attitudes, *Nature Related* nature relatedness, *Engage* behavioral engagement (subscale 23), *CCI* climate change impairment, *Cogem. Impair* cognitive-emotional impairment, *Func. Impair* functional impairment, *Exper* climate change experience, *NA* negative affect, *N* neuroticism, *GAD* generalized anxiety disorder symptoms, *DD* depressive symptoms.

## Study 4: behavioral validation

As a further validation of our scale, we investigated whether CCD and CCI predict pro-environmental behavior (PEB). To account for the problem of heavy use of self-reports and the associated distortions in previous studies investigating CCD-like constructs as predictors of PEB^13,42,43^, we relied on an incentivized social dilemma paradigm^44,45^. We hypothesized that higher levels of CCD and CCI would be associated with a higher probability of showing PEB.

### Method

#### Procedure

The requirement for ethics approval was waived by the IRB because all data in the study were collected in a completely anonymous fashion, and the included constructs were not expected to cause participants undue distress. Data for this study were collected via the German online panel provider *respondi.de* as part of the *Prosocial Personality Project (PPP)*, a large-scale web-based study across six base measurement occasions and several follow-up assessments. More information on the project, including verbatim items, sample sizes, each measurement occasion, and exclusion criteria is available at https://osf.io/m2abp/. At the beginning of the assessment, participants provided informed consent and demographic data. The German version of the CC-DIS was assessed in November of 2021, as was the behavioral paradigm used to measure PEB, the Greater Good Game (GGG)^44^. Following self-report questionnaires, we asked participants to indicate whether they believed the quality of their provided data to be sufficient to be included in our analysis. At the end, participants were debriefed about the goals of this specific measurement wave of the PPP.

#### Participants

The final sample consisted of 494 participants who provided valid answers for the GGG and CDD and CCI items, passed an attention check, answered the quality check positively, and did not provide invalid answers to more than 50% of the scales collected at this measurement occasion. In line with the general a priori exclusion criteria set for the PPP (see https://osf.io/m2abp/), we considered responses to a given scale as invalid if we suspected inattentive response behavior (defined as response times of less than 2 s per item on average as measured with the total time spent on the survey page with the respective items and/or very low variation on that scale, i.e. *SD* = 0). Participants were between 22 and 72 (*M* = 49.76, *SD* = 11.14) years old. All of them identified as cis-gender (37.2% female, 62.8% male), and the majority were native German speakers (96.6%).

#### Measures

For the German version of the CC-DIS, JH and SAK translated the items into German and a bilingual colleague conducted the back-translation into English. Any differences between the original items and the back translation (and resulting consequences for the German items) were resolved in the author team via consensus. For the full list of German items, see Table S21a,b.

The GGG^44^ is a social dilemma paradigm in the style of a nested public goods game. In the game, participants were informed that they were part of an anonymous group of three in which each member was given an endowment and could decide how to spend this endowment. Participants were informed that they would be randomly assigned to groups of three at the end of the data collection and that one trial would be randomly drawn and paid out according to their own and their group members’ decisions. They could choose one of three options: (i) a selfish option, i.e. keeping the endowment for themselves, (ii) a cooperative option, i.e. contributing their endowment to a shared group account which would be doubled by the experimenter and distributed equally across all group members, or (iii) a pro-environmental option, i.e. contributing their endowment to an environmental account which would also be doubled by the experimenter and donated to the German Federation for the Environment and Nature Conservation, a German NGO dedicated to environmental protection. Participants played ten rounds of the GGG with endowments ranging from 0.20€ to 2.00€ (increased in 0.20€-steps), presented in random order.

#### Data analysis

**Figure 2:**
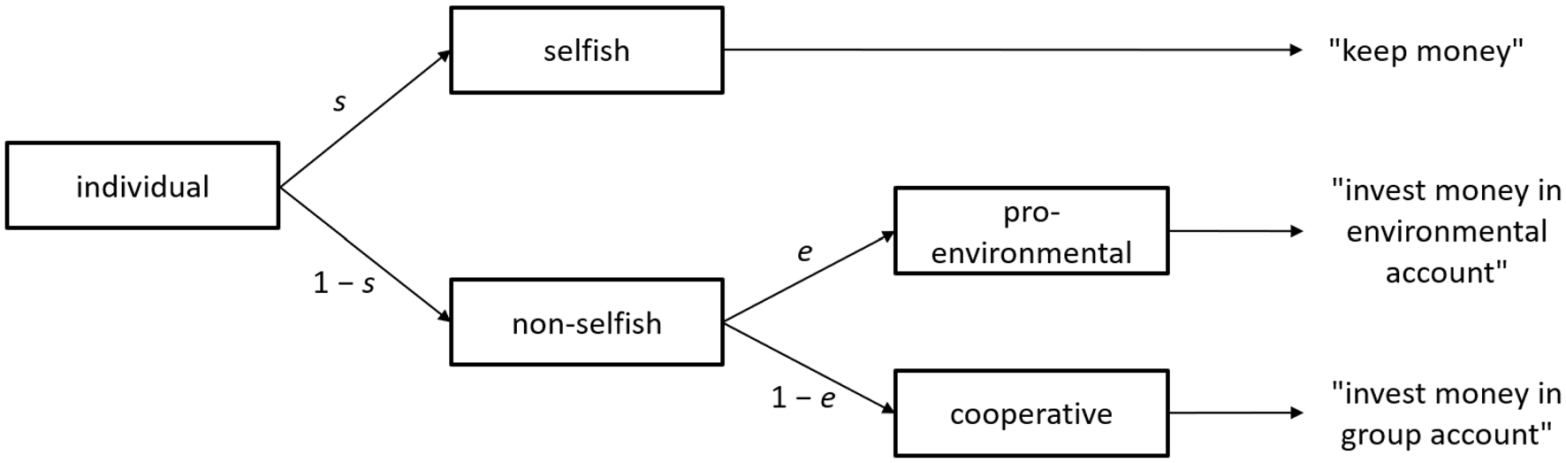
Multinomial processing tree model for the Greater Good Game in Study 4.

GGG data were analyzed using a multinomial processing tree (MPT) model^46,47^ specifically tailored to the GGG^44^ (see Fig. 2). First, parameter *s* was defined as the probability of selfish behavior to distinguish between a selfish and non-selfish choice (probability 1–*s*). Second, parameter *e* was defined as the probability of PEB given non-selfish behavior to distinguish between PEB (probability *e*) and cooperation (probability 1–*e*). In other words, *e* is conditional on *s*. Additionally, we included CCD and CCI as predictors of parameters *s* and *e*. To account for individual differences on these traits and to directly assess their effect on the behavioral parameters *s* and *e*, we fitted a Bayesian hierarchical extension of the MPT model^48^ using the R package TreeBUGS^49^. The model was fitted using standard-normal priors (g-prior) on the regression slopes.

### Results

As this constituted the first application of the German version of the CC-DIS, we first conducted a CFA to determine whether the factor structure replicated (see Table S22a–d). Fit indices suggested acceptable model fit, CFI = 0.903, TLI = 0.888, RMSEA = 0.079, SRMR = 0.081, and excellent factor-specific replicability, and determinacy (*H*_Distress_ = 0.966, *H*_Impairment_ = 1.000, *FD*_Distress_ = 0.983, *FD*_Impairment_ = 1.000). All distress items showed loadings > 0.481 onto the latent distress factor, and all impairment items loadings > 0.432 onto the impairment factor. Internal consistencies were excellent for CCD (α = 0.93, Ω = 0.94) and acceptable for CCI (α = 0.81, Ω = 0.80). Boxplots for the German items are presented in the supplement. Inter-item correlations are presented in Tables S23 and S24.

The most prevalent choice in the GGG was in-group cooperation. Across all trials and participants, 46.6% of decisions were cooperative (22.5% selfish, 31.0% pro-environmental). Parameter estimates for the group means of the MPT parameters (including 95% Bayesian credibility intervals) were *s* = 0.231, 95% CI [0.203; 0.259], and *e* = 0.373, 95% CI [0.339, 0.409].

The regression coefficient of the effect of CCD on *s* was β = − 0.303 with a 95% Bayesian credibility interval of [− 0.390; − 0.213]. The Bayes factor was estimated as BF_10_ > 10,000 (using the Savage-Dickey approximation^54^), indicating very strong support in favor of the hypothesis that higher CCD was associated with a lower probability for selfish behavior. The regression coefficient for the effect of CCD on *e* was β = 0.394 [0.300; 0.481], with BF_10_ > 10,000 indicating very strong support in favor of the hypothesis that higher CCD was associated with a higher probability of PEB.

The regression coefficient for the effect of CCI on *s* was β = 0.035 [− 0.064; 0.134], BF_10_ = 0.176, indicating moderate support in favor of the null hypothesis versus the directed hypothesis that β was larger than zero. Thus, CCI had no substantial effect on selfish behavior. The regression coefficient of the effect of CCI on *e*, however, was β = 0.277 [0.180; 0.370], with BF_10_ > 10,000 indicating very strong support in favor of the hypothesis that higher CCI was associated with a higher probability of PEB.

## Prevalence of CCD and CCI across all studies

**Figure 3:**
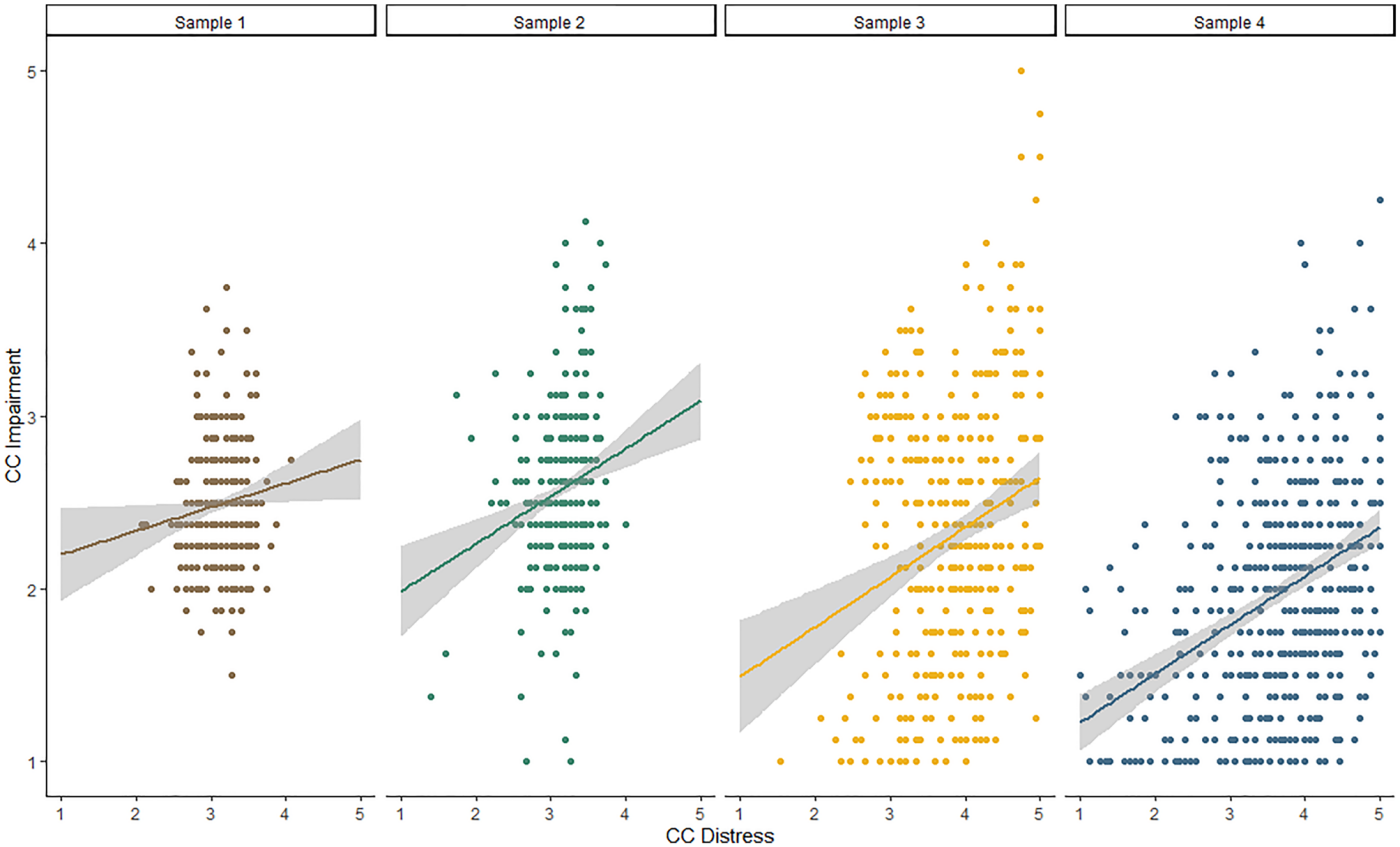
Linear association between CCD and CCI in each of the four samples.

Our third aim was to assess the prevalence of CCD and CCI in different demographic groups. Figure 3 illustrates that CCD and CCI scores showed small to moderate, positive correlations in all studies (*r*_1_ = 0.11, *p* = 0.029; *r*_2_ = 0.21, *p* < 0.001; *r*_3_ = 0.24, *p* < 0.001; *r*_4_ = 0.39, *p* < 0.001), underlining the association in both international (Studies 1–3) and German (Study 4) samples. Mean levels of CCD indicated moderate levels of distress in Studies 1 and 2 (*M*_1_ = 3.14, *SD* = 0.26, *M*_2_ = 3.16, *SD* = 0.30) and moderate to high distress in Studies 3 and 4 (*M*_3_ = 3.84, *SD* = 0.66, *M*_4_ = 3.73, *SD* = 0.90). Average CCI levels were low to moderate and below the scale’s midpoint in all four studies (*M*_1_ = 2.50, *SD* = 0.33; *M*_2_ = 2.58, *SD* = 0.39; *M*_3_ = 2.31, *SD* = 0.77; *M*_4_ = 1.96, *SD* = 0.66).

**Figure 4:**
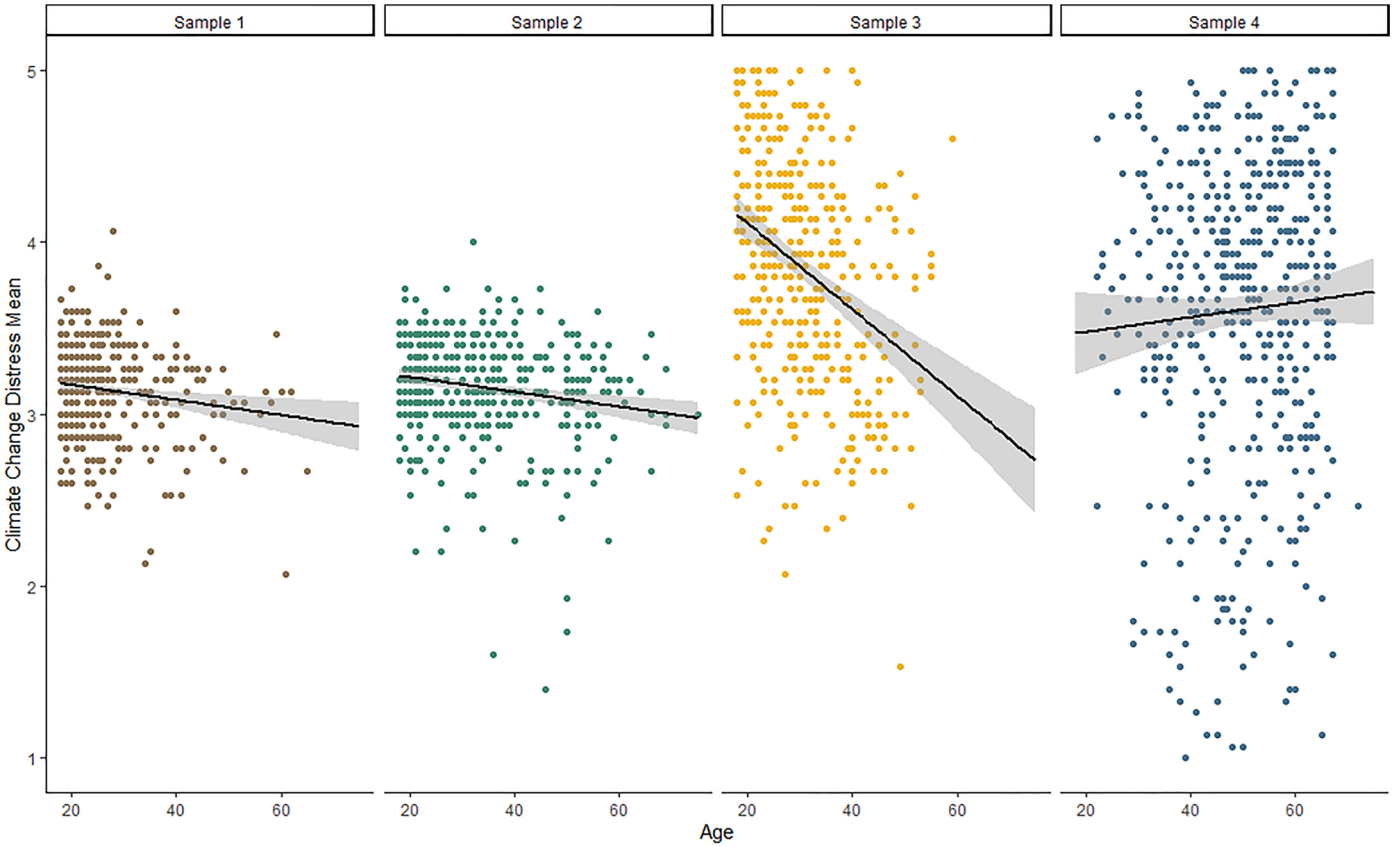
Linear association between CCD and age in each of the four samples.

We hypothesized a negative association of CCD and CCI levels with age, such that younger individuals are more affected. As Fig. 4 illustrates, we observed the expected negative correlation between CCD and age in Studies 1 to 3, but not in Study 4. Effect sizes ranged from small in Study 1 (*r* = − 0.15, *p* = 0.003) and Study 2 (*r* = − 0.18, *p* < 0.001) to medium-sized in Study 3 (*r* = − 0.34, *p* < 0.001). In Study 4, age and CCD were not significantly correlated (*r* = 0.05, *p* = 0.239). For CCI, we observed very small correlations with age in all studies. Correlations were descriptively negative in Studies 1 and 2 (*r*_*1*_ = − 0.11, *p* = 0.024, *r*_*2*_ = − 0.09, *p* = 0.047) and positive in Studies 3 and 4 (*r*_*3*_ = 0.09, *p* = 0.084, *r*_*4*_ = 0.11, *p* = 0.020). Thus, across studies, there appeared to be no consistent age pattern for CCI.

For gender, we compared the level of CCD and CCI between female and male participants. In Studies 1 to 3, women showed significantly higher levels of CCD than men (Study 1: *t* = 2.50, *p* = 0.013, Study 2: *t* = 3.80, *p* < 0.001, Study 3: *t* = 6.36, *p* < 0.001). In the German speaking sample in Study 4, women and men did not differ significantly in their level of CCD (*t* = 0.38, *p* = 0.705). For CCI, we observed no differences between women in men in Studies 1, 2, and 4 (all *p*s > 0.182). In Study 3, contrary to expectations, men showed higher levels of CCI than women (*t* = − 2.89, *p* = 0.004).

## General discussion

Climate change is not only associated with increased rates of psychopathology^26^ but also with substantial levels of (non-pathological) distress in the general population^4^. To monitor the negative mental health effects of climate change and target pathological levels with adequate interventions, it is crucial to distinguish climate change distress from impairment that may indicate a need for intervention. We herein introduced the *Climate Change Distress and Impairment Scale* (CC-DIS), which measures CCD (spanning anxiety, anger, and sadness) and CCI with two distinct subscales (as demonstrated in a series of factor analyses). Across four samples, we observed moderate to high rates of CCD, and low to moderate levels of CCI, underlining the prevalence and gravity of the phenomenon.

Contrary to hypotheses, the demographic patterns were less clear than indicated by previous studies. We observed the hypothesized negative association between CCD and age in the three English-speaking samples^7,18,23–25^ but not in the German-speaking sample, which was part of a rolling panel and therefore unaffected by self-selection bias that may have influenced the composition of the English-speaking samples. However, data for Study 4 was collected less than four months after catastrophic flooding events in the Ahr valley in July 2021, which were heavily covered by national and international media. Thus, awareness of climate change and its consequences was likely still increased among the German general public. This could explain the generally increased levels of CCD in the German-speaking sample. Contrary to our hypothesis, we observed no correlations between CCI and age that were significant at *p* < 0.001. While further replication is needed, this suggests that an exclusive focus on younger individuals may be misguided and that individuals of all ages are at a similar risk for CCI.

Regarding gender, we observed the hypothesized higher levels of CCD in women compared to men in the three English-speaking samples^7,18,50^ but again not in the German-speaking sample. For CCI, we observed no significant patterns in Studies 1, 2, and 4, and higher levels in men in Study 3, which was contrary to hypotheses. We note as an important limitation that we were able to recruit only 38 participants that identified as genderqueer, which precluded us from further investigating CCD and CCI levels in this group. Descriptively, genderqueer participants reported the highest rates of both CCD and CCI, which is in line with the minority stress hypothesis^51^ and highlights the importance of catering future work toward this group. Additional large-scale studies will be needed to investigate age and gender effects on CCD and CCI in different countries and determine whether the observed CCD results indicate cultural differences.

Beyond sampling in different countries and recruiting participants of all ages and genders, future work should pay special attention to ensuring ethnic, racial, and socioeconomic diversity of the samples, as all of these are likely correlates of both climate change exposure as well as CCD and CCI. At the country level, future work should refer to the United Nations’ environmental vulnerability index and environmental performance index to quantify exposure to climate change and climate protection efforts and sample from countries with diverse scores on these indices.

### Validity

In a series of factor analyses, we aimed to establish structural validity of the scale. We constructed the CC-DIS aiming for an equal number of positively and negatively worded items. Empirically, we observed that the inclusion of negatively worded items resulted in poorer overall model fit, which required the introduction of a method factor to adjust for this additional source of systematic but theoretically uninteresting source of variability in order to more appropriately represent the underlying constructs the scale set out to measure. Following this, we observed TLI scores slightly below 0.90 for Studies 2 and 4, which is traditionally considered a cutoff for adequate model fit (CFI, RMSEA, and SRMR were all in the range considered acceptable or good). It is possible that the low TLI scores are the result of increased model complexity due to the method factor, as TLI places more emphasis on parsimony than the other fit indices. Additional work is needed to further validate the scale and replicate or modify the factor solution presented herein. However, we do note that the consistency of the estimated factor structure of the CC-DIS across Studies 1–4 was moderate (*ICC* = 0.703, 95% CI [0.555, 0.811]) providing initial evidence that the distress and impairment constructs are relatively stable in their representation across independent samples.

In addition to structural validity, we aimed to establish external validity of the CC-DIS. In Study 3, CCD and CCI showed convergent validity, as CCD correlated most strongly with distress about the consequences of climate change^16^ and CCI with cognitive-emotional and functional impairment as measured by the climate anxiety scale^18^. Interestingly, CCI also showed a large correlation with the climate change experience subscale, suggesting that individuals who had personally experienced more of the consequences of climate change were more impaired by it. Both CCD and CCI also showed excellent discriminant validity in the clinical domain, indicating that CCD measures distress that is specific to climate change and not just a manifestation of general negative affect or clinical levels of negative affect as they occur during depression or generalized anxiety disorder. CCI also proved to be distinct from psychopathology and from a generally low quality of life.

In the environmental domain, the picture was less clear. CCI showed the expected discriminant validity, indicated by a null correlation with environmental attitudes and a moderate correlation with nature relatedness. CCD, in contrast, showed large correlations with both constructs, indicating that individuals who feel closer to nature were more distressed by its destruction. Additionally, the scale used to assess environmental attitudes^41^ focuses on attitudes surrounding the reality of growth limits, the fragility of nature’s balance, the possibility of an ecological crisis, etc. It could thus even be used as a proxy for a participant's level of awareness of and care about climate change. Therefore, it is also not surprising that CCD correlated substantially with this scale, but further investigations into the nature of this association are needed.

In Study 4, we demonstrated that CCD is predictive of PEB in the GGG^44^, a social dilemma in which participants have to decide between selfish, cooperative, and pro-environmental behavior. This finding replicates previous work demonstrating a positive association between CCD-like constructs and self-reported PEB^52–54^ and provides an important extension in that it measures actual behavior where previous work focused on behavioral intentions that often overestimate PEB^55^. The finding is also in line with data from Study 3, where we observed a large positive correlation between CCD and the behavioral engagement subscale in the climate anxiety scale^18^. CCD could thus be seen as an adaptive response to climate change that may even be pivotal in mitigating its negative effects. To further probe the CCD-PEB association, future work should attempt to experimentally induce different state-levels of CCD and measure subsequent PEB levels. Additionally, as past work has showed that different types of climate change associated affect drive different types of behavior^10,12,14,15^, future work is needed to compare whether CCD predicts different types or a broader set of behavior than, for example, climate anxiety.

In addition to CCD, we also found CCI to be predictive of PEB. At first glance, this finding may seem counter-intuitive, as impairment typically precludes goal-directed behavior and should thus predict lower PEB. However, this may be restricted to behavior that is effortful or demanding in some other aspect. For instance, making sustainable choices when buying groceries requires cognitive and time resources that someone high on impairment may not have. In contrast, PEB as measured by economic games or social dilemmas such as the GGG is relatively effortless. Future work is need to probe the impairment-PEB association, for instance by manipulating the level of effort and personal cost the PEB requires, or by measuring naturally occurring PEB in daily life and putting that in context to the level of CCD and CCI.

### Conclusion

We introduced the *climate change distress and impairment scale (CC-DIS)*, which is able to distinguish the affective experience of distress over climate change (covering anger, anxiety, and sadness) from functional impairment. Across four studies, we observed moderate to high levels of CCD, underlining that CCD is highly prevalent in the general population. At the same time, we observed only low to moderate levels of CCI, which underlines that most individuals are not significantly impaired by their experience of CCD. In Study 4, we found very strong support for the hypothesis that higher CCD and CCI are associated with a higher probability for PEB. This underlines that affective responses to climate change are important drivers of efforts to reduce the negative impact of climate change and deserve scientific attention. Future work with large-scale, multinational, and diverse samples is needed to further quantify the prevalence of CCD and CCI and characterize the subgroup of individuals with high or very high levels of CCI.

## Supplementary Information

Supplementary Table S1.

Supplementary Table S2.

Supplementary Table S3.

Supplementary Table S4.

Supplementary Table S5.

Supplementary Table S6.

Supplementary Table S7.

Supplementary Table S8.

Supplementary Table S9.

Supplementary Table S10.

Supplementary Table S11.

Supplementary Table S12.

Supplementary Table S13.

Supplementary Table S14.

Supplementary Table S15.

Supplementary Table S16.

Supplementary Table S17.

Supplementary Table S18.

Supplementary Table S19.

Supplementary Table S20.

Supplementary Table S21.

Supplementary Table S22.

Supplementary Table S23.

Supplementary Table S24.

## Data Availability

Data and analysis code for all four studies are openly available in our osf online repository (https://osf.io/eprdw/) in the folder “Climate Change Distress and Impairment Scale (CC-DIS)”, subfolder “Data and analysis code”. The full list of English and German items is also accessible via this link.
