# Supplementary material for: Introduction and behavioral validation of the climate change distress and impairment scale
Source: Sci Rep. 2023 Jul 12;13:11272. doi: 10.1038/s41598-023-37573-4 (PMC10338517; doi:10.1038/s41598-023-37573-4)
Supplement: Supplementary file 1 — Supplementary Table S1. [file 41598_2023_37573_MOESM1_ESM.pdf]

**Table S1**

*Study 1 list of administered items (initial item pool, 84 items)*

| Anger |
|---|
| Unsustainable behavior bothers me. |
| I am not mad when others damage the climate. |
| I do not judge others for their environmental behavior. |
| I do not care about others wasting energy. |
| It is okay that some people are not interested in climate change. |
| I feel good about the steps that have already been taken to address climate change. |
| I do not get upset when others ignore climate change. |
| I am calm because I think that enough is being done about climate change. |
| I resent others for not behaving pro-environmentally. |
| It makes me furious when others deny climate change. |
| I get upset when others behave unsustainably. |
| I am enraged that we have missed many chances to stop climate change. |
| I want companies who destroy the climate to be punished. |
| I feel aggressive towards people who destroy the environment. |
| I feel angry when I see how little is done to combat climate change. |
| I am not angry that some countries have missed their climate protection goals. |

*Note.* Table is continued on the next page.

**Table S1 Continued**

*Study 1 list of administered items (initial item pool, 84 items)*

| Anxiety |
| --- |
| I feel tense when I think about climate change. |
| I do not worry about the effects of climate change. |
| I find climate change harmless. |
| Despite climate change, I look positively toward the future. |
| I am not worried about climate change. |
| Climate change does not scare me. |
| I believe that we will get climate change under control. |
| I feel carefree when I think about climate change. |
| When I think about climate change, I worry about the future. |
| I fear that we cannot stop climate change. |
| I fear that some resources will become scarce because of climate change. |
| I fear that political conflicts will increase due to climate change. |
| I fear that more animal species will go extinct. |
| The uncertainty about how climate change will progress scares me. |
| I am scared that people will lose their homes because of climate change. |
| I do not fear for my future on this planet. |

*Note.* Table is continued on the next page.

**Table S1 Continued**

*Study 1 list of administered items (initial item pool, 84 items)*

| Guilt |
|---|
| I am ashamed that I do not behave more sustainably. |
| I do enough to counteract climate change. |
| I am proud of my environmentally conscious behavior. |
| I am not responsible for stopping climate change. |
| I am happy with my contribution to climate protection. |
| I am comfortable not making sacrifices for climate protection. |
| My commitment to climate protection is sufficient. |
| Climate change is not my problem. |
| Humanity is destroying the planet with its self-interest. |
| I feel that I am not doing enough to stop climate change. |
| Sometimes I think about how my actions have harmed the climate. |
| I think climate change is my fault too. |
| I feel bad when I waste energy. |
| When I see news about climate change, I feel complicit. |
| I am embarrassed that I used to take climate change less seriously than I do now. |
| Others are to blame for climate change. |

*Note*. Table is continued on the next page.

**Table S1 Continued**

*Study 1 list of administered items (initial item pool, 84 items)*

| Sadness |
| --- |
| News about climate change makes me feel depressed. |
| The extinction of animal species does not make me feel sad. |
| I am not concerned about climate change. |
| I find forecasts about climate change amusing. |
| I feel happy when I think about the current state of the environment. |
| I am happy about the changes that climate change brings about. |
| I am not troubled by news about the consequences of climate change. |
| I am not sad about climate change. |
| Thinking about climate change makes me want to cry. |
| I feel sad when someone mentions climate change. |
| Climate change makes me feel hopeless. |
| I feel sad that climate change is causing people and animals to suffer. |
| I feel sad that some parts of the environment will not recover from the effects of climate change. |
| The impact that climate change has had on the planet saddens me. |
| I feel miserable when I think about climate change. |

*Note*. Table is continued on the next page.

**Table S1 Continued**

*Study 1 list of administered items (initial item pool, 84 items)*

| Impairment |
| --- |
| Climate change drains all my energy. |
| My thoughts and feelings about climate change do not affect how well I sleep. |
| When I think about climate change, I get a headache or stomachache. |
| Because of climate change, I am overwhelmed by everyday activities. |
| When I think about climate change I do not feel like doing anything. |
| I have given up hobbies because they are bad for the climate. |
| Climate change does not keep me from enjoying my life. |
| My thoughts and feelings about climate change do not negatively impact my everyday life. |
| I have no trouble mentally tuning out climate change. |
| I can easily balance my commitment to climate protection with my everyday life. |
| I fight with family and friends because they see climate change differently than I do. |
| I have lost friends because we had different opinions on climate change |
| Constant discussions about climate change are affecting my relationships. |
| I have never broken ties with anyone because of a differing opinion on climate change. |
| I do not feel excluded by others because of my views on climate change. |
| It is easy for me to find friends who see climate change the way I do. |
