# Supplementary material for: Introduction and behavioral validation of the climate change distress and impairment scale
Source: Sci Rep. 2023 Jul 12;13:11272. doi: 10.1038/s41598-023-37573-4 (PMC10338517; doi:10.1038/s41598-023-37573-4)
Supplement: Supplementary file 2 — Supplementary Table S2. [file 41598_2023_37573_MOESM2_ESM.pdf]

**Table S2**

*Demographic data for participants in studies 1, 2, and 3.*

| | **Study 1** *N* = 384 | | **Study 2** *N* = 447 | | **Study 3** *N* = 374 | |
|---|---|---|---|---|---|---|
| | *n* | % | *n* | % | *n* | % |
| **Gender** | | | | | | |
| Female | 151 | 39.32 | 236 | 52.80 | 188 | 50.27 |
| Male | 224 | 58.33 | 186 | 41.61 | 174 | 46.52 |
| Transgender | - | - | 3 | 0.67 | 2 | 0.53 |
| Non-binary | 5 | 1.30 | 18 | 4.03 | 10 | 2.67 |
| Gender-neutral | 3 | 0.78 | 2 | 0.45 | - | - |
| Other | - | - | 1 | 0.22 | - | - |
| Did not want to disclose | 1 | 0.26 | 1 | 0.22 | - | - |
| **Race** | | | | | | |
| White | 340 | 88.54 | 350 | 78.30 | 185 | 49.47 |
| Black | 12 | 3.12 | 41 | 9.17 | 80 | 21.39 |
| Asian | 11 | 2.86 | 23 | 5.15 | 41 | 10.96 |
| Mixed-racial | 10 | 2.60 | 18 | 4.03 | 44 | 11.76 |
| Other | 5 | 1.30 | 11 | 2.46 | 18 | 4.81 |
| Did not want to disclose | - | - | 4 | 0.89 | - | - |
| missing | 6 | 1.56 | - | - | 6 | 1.6 |
| **English language proficiency** | | | | | | |
| Fluent | 320 | 83.33 | - | - | - | - |
| Native | 64 | 16.67 | 447 | 100.00 | 374 | 100.00 |
| **Education** | | | | | | |
| no degree | - | - | 6 | 1.34 | 9 | 2.41 |
| None (still in school) | 9 | 2.34 | 5 | 1.12 | 9 | 2.41 |
| Junior high school | 8 | 2.08 | 1 | 0.22 | 1 | 0.27 |
| High school | 143 | 37.24 | 150 | 33.56 | 115 | 30.75 |
| Undergraduate degree | 123 | 32.03 | 179 | 40.04 | 132 | 35.29 |
| Graduate degree | 82 | 21.35 | 82 | 18.34 | 94 | 25.13 |
| PhD | 11 | 2.86 | 20 | 4.47 | 7 | 1.87 |
| Did not want to disclose | 8 | 2.08 | 4 | 0.89 | 7 | 1.87 |

*Note*. Table is continued on the next page.

**Table S2 Continued**

*Demographic data for participants in studies 1, 2, and 3.*

| | **Study 1** *N* = 384 | | **Study 2** *N* = 447 | | **Study 3** *N* = 374 | |
|---|---|---|---|---|---|---|
| | *n* | % | *n* | % | *n* | % |
| **Occupation** | | | | | | |
| Unemployed or job seeking | 64 | 16.67 | 40 | 8.95 | 31 | 8.29 |
| Student (High school) | 36 | 9.38 | 2 | 0.45 | 3 | 0.80 |
| Student (College/University/apprentice) | 118 | 30.73 | 80 | 17.90 | 62 | 16.58 |
| (Self-)Employed | 156 | 40.62 | 289 | 64.65 | 268 | 71.66 |
| Homemaker | 3 | 0.78 | 17 | 3.80 | 7 | 1.87 |
| Retired | 1 | 0.26 | 13 | 2.91 | - | - |
| Other | 2 | 0.52 | 5 | 1.12 | 2 | 0.53 |
| Did not want to disclose | 4 | 1.04 | 1 | 0.22 | 1 | 0.27 |
| **Monthly net income** | | | | | | |
| < 500 USD | 135 | 35.16 | 82 | 18.34 | 54 | 14.44 |
| 500-1000 USD | 71 | 18.49 | 51 | 11.41 | 40 | 10.70 |
| 1000-1500 USD | 48 | 12.5 | 41 | 9.17 | 33 | 8.82 |
| 1500-2000 USD | 30 | 7.81 | 72 | 16.11 | 66 | 17.65 |
| 2000-3000USD | 27 | 7.03 | 83 | 18.57 | 60 | 16.04 |
| > 3000 USD | 18 | 4.69 | 67 | 14.99 | 73 | 19.52 |
| Did not want to disclose | 55 | 14.32 | 51 | 11.41 | 48 | 12.83 |
| **Mental health diagnosis** | | | | | | |
| Yes | 81 | 21.09 | 177 | 39.60 | 93 | 24.87 |
| No | 288 | 75.00 | 264 | 59.06 | 275 | 73.53 |
| Not sure | 10 | 2.60 | 4 | 0.89 | 3 | 0.80 |
| Did not want to disclose | 5 | 1.30 | 2 | 0.45 | 3 | 0.80 |
| **Treatment** | | | | | | |
| Yes | 76 | 19.79 | 163 | 36.47 | 94 | 25.13 |
| No | 303 | 78.91 | 278 | 62.19 | 279 | 74.60 |
| Not sure | 1 | 0.26 | 4 | 0.89 | - | - |
| Did not want to disclose | 4 | 1.04 | 2 | 0.45 | 1 | 0.27 |
| **Medication** | | | | | | |
| Yes | 84 | 21.88 | 149 | 33.33 | 90 | 24.06 |
| No | 295 | 76.82 | 294 | 65.77 | 281 | 75.13 |
| Not sure | 3 | 0.78 | 1 | 0.22 | 2 | 0.53 |
| Did not want to disclose | 2 | 0.52 | 3 | 0.67 | 1 | 0.27 |

*Note*. Mental health diagnosis = Participants self-reported whether they had ever received a mental health diagnosis by a health professional (e.g. a GP, psychiatrist, psychologist). Treatment = Participants self-reported whether they had ever received professional treatment for a mental health diagnosis. Medication = Participants self-reported whether they had ever taken prescribed medication to treat a mental health diagnosis. Table is continued on the next page.

**Table S2 Continued**

*Demographic data for participants in studies 1, 2, and 3.*

| | **Study 1** *N* = 384 | | **Study 2** *N* = 447 | | **Study 3** *N* = 374 | |
|---|---|---|---|---|---|---|
| | *n* | % | *n* | % | *n* | % |
| **Country of residence** | | | | | | |
| Australia | 2 | 0.52 | 5 | 1.12 | 1 | 0.27 |
| Austria | 2 | 0.52 | - | - | - | - |
| Belgium | 4 | 1.04 | - | - | - | - |
| Canada | 1 | 0.26 | 57 | 12.75 | 35 | 9.36 |
| Czech Republic | 1 | 0.26 | - | - | - | - |
| Estonia | 5 | 1.30 | - | - | - | - |
| Finland | 6 | 1.56 | - | - | - | - |
| France | 2 | 0.52 | - | - | - | - |
| Germany | 6 | 1.56 | - | - | - | - |
| Greece | 20 | 5.21 | - | - | - | - |
| Hungary | 8 | 2.08 | 1 | 0.22 | - | - |
| Ireland | 2 | 0.52 | 31 | 6.94 | 3 | 0.80 |
| Israel | 2 | 0.52 | - | - | - | - |
| Italy | 38 | 9.90 | - | - | 1 | 0.27 |
| Japan | 2 | 0.52 | - | - | - | - |
| Latvia | 4 | 1.04 | - | - | - | - |
| México | 2 | 0.52 | - | - | - | - |
| Netherlands | 10 | 2.60 | - | - | 1 | 0.27 |
| New Zealand | 2 | 0.52 | 3 | 0.67 | 6 | 1.60 |
| Norway | 2 | 0.52 | - | - | - | - |
| Poland | 112 | 29.2 | - | - | - | - |
| Portugal | 72 | 18.8 | - | - | - | - |
| Slovenia | 2 | 0.52 | - | - | - | - |
| South Africa | 20 | 5.21 | 11 | 2.46 | 26 | 6.95 |
| Spain | 16 | 4.17 | 1 | 0.22 | - | - |
| Sweden | 2 | 0.52 | - | - | - | - |
| Ukraine | - | - | - | - | 1 | 0.27 |
| United Kingdom | 32 | 8.33 | 143 | 31.99 | 159 | 42.51 |
| United States | 7 | 1.82 | 194 | 43.40 | 128 | 34.22 |
| Did not want to disclose | - | - | 1 | 0.22 | 13 | 3.48 |
