# Supplementary material for: Introduction and behavioral validation of the climate change distress and impairment scale
Source: Sci Rep. 2023 Jul 12;13:11272. doi: 10.1038/s41598-023-37573-4 (PMC10338517; doi:10.1038/s41598-023-37573-4)
Supplement: Supplementary file 9 — Supplementary Table S9. [file 41598_2023_37573_MOESM9_ESM.pdf]

Table S9

Study 1 EFA factor loading matrix for the five factor solution.

	Factor 1	Factor 2	Factor 3	Factor 4	Factor 5
SS loadings	9.94	7.38	3.50	2.54	1.33
Proportion Var	.17	.13	.06	.04	.02
Cumulative Var	.17	.30	.37	.41	.43

Note. Test of the hypothesis that five factors are sufficient. The chi square statistic is 2016.7 on 1321 degrees of freedom, p = 5.7e-32. SS loadings = sum of squared loadings; Proportion Var = proportion variance explained; Cumulative Var = cumulative variance explained.

Table S9

Study 1 EFA factor loading matrix for the five factor solution.

Items	Factor 1	Factor 2	Factor 3	Factor 4	Factor 5
ang1	.44				
ang2				.47	
ang3	.48			.41	
ang4	.46				
ang5	.40				
ang6	.43			.42	
ang7				.49	
ang8	.48			.40	
ang9_r	.57			.35	
ang10_r	.66				
ang11_r	.47			.40	
ang12_r	.59				
ang13_r	.58				
ang15_r	.64				
ang16_r	.57				
anx2	.53		.35		
anx3			.51		
anx4	.45		.41		
anx5			.36		
anx6	.41		.47		
anx7	.49		.47		
anx8	.41		.54		
anx9_r	.43				
anx10_r	.71				
anx13_r	.72				
anx14_r	.62				
anx16_r	.62				
guilt5					
guilt6	.53				
guilt7			.41		
guilt8		.36			

Note. Table is continued on the next page for items assessing sadness and impairment.

Table S9 Continued

Study 1 EFA factor loading matrix for the five factor solution.

Items	Factor 1	Factor 2	Factor 3	Factor 4	Factor 5
sad1	.43		.35		
sad2		.57			.61
sad3		.36			
sad4		.39	.37		.36
sad5	.50		.42		
sad6	.57		.39		
sad7	.51		.49		
sad8		.50			
sad13_r	.64				
sad14_r	.41				
sad16_r	.74				
impg1		.69			
impg2		.61			
impg3		.73			
impg6_r		.53			
impg7_r		.60			
impg8_r		.59			
impg10_r		.65			
imps1		.41		.43	
imps3		.60			
imps4_r		.45			
imps5_r		.46			
impw1		.64			
impw2		.77			
impw3_r		.67			
impw4_r		.66			
