# Supplementary material for: Introduction and behavioral validation of the climate change distress and impairment scale
Source: Sci Rep. 2023 Jul 12;13:11272. doi: 10.1038/s41598-023-37573-4 (PMC10338517; doi:10.1038/s41598-023-37573-4)
Supplement: Supplementary file 10 — Supplementary Table S10. [file 41598_2023_37573_MOESM10_ESM.pdf]

Table S10

Study 1 EFA factor loading matrix for the two factor solution.

	Factor 1	Factor 2
SS loadings	13.05	8.24
Proportion Var	.23	.14
Cumulative Var	.23	.37

Note. Test of the hypothesis that two factors are sufficient. The chi square statistic is 2870.09 on 1483 degrees of freedom, p = 4.16e-91. Overall explained variance only 37%. Factor 1: negative climate affect (anger, anxiety, sadness, guilt). Factor 2: impairment (and cross-loadings from several sadness items that could denote depression like constructs). SS loadings = sum of squared loadings; Proportion Var = proportion variance explained; Cumulative Var = cumulative variance explained.

Table S10

Study 1 EFA factor loading matrix for the two factor solution.

Items	Factor 1	Factor 2
ang1	.52	
ang2		
ang3	.63	
ang4	.56	
ang5	.56	
ang6	.57	
ang7	.37	
ang8	.63	
ang9_r	.60	
ang10_r	.60	
ang11_r	.50	
ang12_r	.52	
ang13_r	.52	
ang15_r	.67	
ang16_r	.63	
anx2	.65	
anx3		
anx4	.58	
anx5	.46	
anx6	.58	
anx7	.69	
anx8	.60	
anx9_r	.45	
anx10_r	.72	
anx13_r	.71	
anx14_r	.71	
anx16_r	.63	
guilt5		
guilt6	.53	
guilt7	.37	
guilt8		.36

Note. Table is continued on the next page for items assessing sadness and impairment.

Table S10 Continued

Study 1 EFA factor loading matrix for the two factor solution.

	Factor 1	Factor 2
sad1	.56	
sad2		.70
sad3	.43	.46
sad4	.41	.51
sad5	.60	
sad6	.67	
sad7	.69	
sad8	.38	.57
sad13_r	.68	
sad14_r	.40	
sad16_r	.70	
impg1		.73
impg2		.69
impg3		.79
impg6_r		.46
impg7_r		.58
impg8_r		.53
impg10_r		.63
imps1		.45
imps3		.62
imps4_r		.44
imps5_r		.43
impw1		.68
impw2		.79
impw3_r		.65
impw4_r		.60
