# Supplementary material for: Introduction and behavioral validation of the climate change distress and impairment scale
Source: Sci Rep. 2023 Jul 12;13:11272. doi: 10.1038/s41598-023-37573-4 (PMC10338517; doi:10.1038/s41598-023-37573-4)
Supplement: Supplementary file 11 — Supplementary Table S11. [file 41598_2023_37573_MOESM11_ESM.pdf]

Table S11

Study 2 list of administered items (including new set of guilt items).

Anger
I feel angry when I see how little is done to combat climate change.
I am not angry that some countries have missed their climate protection goals.
I do not care about others wasting energy.
I do not judge others for their environmental behavior.
I resent others for not behaving pro-environmentally.
I am not mad when others damage the climate.
It is okay that some people are not interested in climate change.
I get upset when others behave unsustainably.
I am calm because I think that enough is being done about climate change.
I feel aggressive towards people who destroy the environment.
It makes me furious when others deny climate change.
I am enraged that we have missed many chances to stop climate change.
I want companies who destroy the climate to be punished.
Unsustainable behavior bothers me.
I do not get upset when others ignore climate change.

Note. Table is continued on the next page.

Table S11 Continued

Study 2 list of administered items (including new set of guilt items).

Anxiety
I fear that some resources will become scarce because of climate change.
I do not worry about the effects of climate change.
I am not worried about climate change.
I feel carefree when I think about climate change.
When I think about climate change, I worry about the future.
I fear that we cannot stop climate change.
I fear that political conflicts will increase due to climate change.
The uncertainty about how climate change will progress scares me.
I fear that more animal species will go extinct.
I am scared that people will lose their homes because of climate change.
I do not fear for my future on this planet.
Climate change does not scare me.

Note. Table is continued on the next page.

Table S11 Continued

Study 2 list of administered items (including new set of guilt items).

Guilt
When I see news about climate change, I feel complicit.
I do not feel guilty about having hobbies that harm the climate.
I do not feel guilty about putting my own needs before climate protection.
I do not feel guilty when I use a lot of water.
I am not to blame for the state of the environment.
I am not to blame for the consequences of climate change.
I feel bad when I waste energy.
I think climate change is my fault too.
I feel guilty because I do not do enough to educate myself about climate change.
I feel guilty because I produce more greenhouse gases than I need to.
I feel guilty because I could do more to save natural resources.
I feel guilty because the lifestyle I want is not sustainable.
I do not feel guilty about producing waste
I do not feel guilty about using up a lot of resources.

Note. Table is continued on the next page.

Table S11 Continued

Study 2 list of administered items (including new set of guilt items).

Sadness
News about climate change makes me feel depressed.
I am not sad about climate change.
I am happy about the changes that climate change brings about.
Thinking about climate change makes me want to cry.
I feel sad when someone mentions climate change.
Climate change makes me feel hopeless.
I feel sad that climate change is causing people and animals to suffer.
I feel sad that some parts of the environment will not recover from the effects of climate change.
The impact that climate change has had on the planet saddens me.
I feel miserable when I think about climate change.

Note. Table is continued on the next page.

Table S11 Continued

Study 2 list of administered items (including new set of guilt items).

Impairment
Climate change drains all my energy.
When I think about climate change, I get a headache or stomachache.
Because of climate change, I am overwhelmed by everyday activities.
My thoughts and feelings about climate change do not affect how well I sleep.
My thoughts and feelings about climate change do not negatively impact my everyday life.
I have no trouble mentally tuning out climate change.
Climate change does not keep me from enjoying my life.
I fight with family and friends because they see climate change differently than I do.
Constant discussions about climate change are affecting my relationships.
I have never broken ties with anyone because of a differing opinion on climate change.
I do not feel excluded by others because of my views on climate change.
When I think about climate change, I cannot bring myself to work/study.
I frequently think about climate change instead of working/studying.
Even when I think about climate change, I can easily concentrate at work/in school.
On days on which I worry about climate change, I am as productive as usual.
