# Supplementary material for: Introduction and behavioral validation of the climate change distress and impairment scale
Source: Sci Rep. 2023 Jul 12;13:11272. doi: 10.1038/s41598-023-37573-4 (PMC10338517; doi:10.1038/s41598-023-37573-4)
Supplement: Supplementary file 12 — Supplementary Table S12. [file 41598_2023_37573_MOESM12_ESM.pdf]

Table S12

Study 2 EFA factor loading matrix for the five factor solution.

	Factor 1	Factor 2	Factor 3	Factor 4	Factor 5
SS loadings	12.24	7.35	5.46	3.46	2.21
Proportion Var	.18	.11	.08	.05	.03
Cumulative Var	.18	.29	.37	.43	.46

Note. Test of the hypothesis that 5 factors are sufficient. The chi square statistic is 4009.23 on 1886 degrees of freedom, p = 7.22e-155. SS loadings = sum of squared loadings; Proportion Var = proportion variance explained; Cumulative Var = cumulative variance explained.

Table S12

Study 2 EFA factor loading matrix for the five factor solution.

Items	Factor 1	Factor 2	Factor 3	Factor 4	Factor 5
ang1	.47				
ang2	.48				
ang3	.71				
ang4	.49				
ang5	.70				
ang6	.60				
ang7	.45				
ang8	.76				
ang9_r	.55				
ang10_r	.54		.36		
ang11_r			.40		
ang12_r	.39		.49		
ang13_r	.44		.38		
ang15_r	.53		.50		
ang16_r	.42				.56
anx2	.67				
anx3	.35				
anx4	.63				
anx5	.48				
anx6	.67				
anx7	.65				
anx8	.55				
anx9_r	.42		.37		
anx10_r	.51		.44		
anx13_r	.55		.36		
anx14_r	.60		.43		
anx16_r	.45				.51
ang1	.47				
ang2	.48				
ang3	.71				
ang4	.49				

Note. Table is continued on the next page for items assessing sadness and guilt.

Table S12 Continued

Study 2 EFA factor loading matrix for the five factor solution.

Items	Factor 1	Factor 2	Factor 3	Factor 4	Factor 5
sad1	.60				
sad2	.48	.50			
sad3	.55				
sad4	.55				
sad5	.64				
sad6	.70				
sad7	.67				
sad8	.59				
sad13_r	.37				.45
sad14_r					.45
sad16_r	.59		.40		
guilt5				.61	
guilt6			.38		
guilt7				.42	
guilt17_r			.49	.36	
guilt18_r			.57	.39	
guilt19				.64	
guilt20_r			.65		
guilt21_r			.62		
guilt22				.60	
guilt23_r			.62		
guilt24				.54	
guilt25_r			.60		
guilt26_r			.57		
guilt27				.56	

Note. Table is continued on the next page for items assessing impairment.

Table S12 Continued

Study 2 EFA factor loading matrix for the five factor solution.

Items	Factor 1	Factor 2	Factor 3	Factor 4	Factor 5
impg1		.76			
impg2		.72			
impg3		.78			
impg6_r		.66			
impg7_r		.68			
impg8_r		.57			
impg10_r		.67			
imps1		.50			
imps3		.57			
imps4_r		.36			
imps5_r		.41			
impw1		.71			
impw2		.69			
impw3_r		.61			
impw4_r		.68			
