# Supplementary material for: Introduction and behavioral validation of the climate change distress and impairment scale
Source: Sci Rep. 2023 Jul 12;13:11272. doi: 10.1038/s41598-023-37573-4 (PMC10338517; doi:10.1038/s41598-023-37573-4)
Supplement: Supplementary file 13 — Supplementary Table S13. [file 41598_2023_37573_MOESM13_ESM.pdf]

Table S13

Study 2 EFA factor loading matrix for the two factor solution (full item set).

	Factor 1	Factor 2
SS loadings	15.43	7.84
Proportion Var	.23	.12
Cumulative Var	.23	.35

Note. Test of the hypothesis that two factors are sufficient. The chi square statistic is 6858.62 on 2078 degrees of freedom, $p = 0$. SS loadings = sum of squared loadings; Proportion Var = proportion variance explained; Cumulative Var = cumulative variance explained.

Table S13

Study 2 EFA factor loading matrix for the two factor solution (full item set).

Item	Factor 1	Factor 2
ang1	.46	
ang2	.51	
ang3	.69	
ang4	.47	
ang5	.67	
ang6	.51	
ang7	.35	
ang8	.74	
ang9_r	.60	
ang10_r	.59	
ang11_r	.43	
ang12_r	.54	
ang13_r	.56	
ang15_r	.67	
ang16_r	.52	
anx2	.71	
anx3		
anx4	.62	
anx5	.50	
anx6	.68	
anx7	.69	
anx8	.57	
anx9_r	.55	
anx10_r	.65	
anx13_r	.67	
anx14_r	.75	
anx16_r	.61	

Note. Table is continued on the next page for items assessing sadness and guilt.

Table S13 Continued

Study 2 EFA factor loading matrix for the two factor solution (full item set).

Item	Factor 1	Factor 2
sad1	.65	
sad2	.51	.50
sad3	.59	
sad4	.56	
sad5	.67	
sad6	.67	
sad7	.68	
sad8	.65	.37
sad13_r	.45	
sad14_r	.43	
sad16_r	.74	
guilt5	.35	
guilt6	.36	
guilt7		
guilt17_r		
guilt18_r		
guilt19	.37	
guilt20_r	.46	
guilt21_r	.45	
guilt22		
guilt23_r	.41	
guilt24		
guilt25_r	.48	
guilt26_r	.36	
guilt27	.40	

Note. Table is continued on the next page for items assessing impairment.

Table S13 Continued

Study 2 EFA factor loading matrix for the two factor solution (full item set).

Item	Factor 1	Factor 2
impg1		.77
impg2		.73
impg3		.81
impg6_r		.65
impg7_r		.64
impg8_r		.55
impg10_r		.65
imps1		.56
imps3		.62
imps4_r		.38
imps5_r		.42
impw1		.75
impw2		.71
impw3_r		.53
impw4_r		.60
