# Supplementary material for: Introduction and behavioral validation of the climate change distress and impairment scale
Source: Sci Rep. 2023 Jul 12;13:11272. doi: 10.1038/s41598-023-37573-4 (PMC10338517; doi:10.1038/s41598-023-37573-4)
Supplement: Supplementary file 16 — Supplementary Table S16. [file 41598_2023_37573_MOESM16_ESM.pdf]

Table S16

Study 2 EFA factor loading matrix for the two factor solution (reduced item set).

	Factor 1	Factor 2
SS loadings	10.18	6.32
Proportion Var	.28	.18
Cumulative Var	.28	.46

Note. Test of the hypothesis that two factors are sufficient. The chi square is 2141.78 on 559 degrees of freedom, p = 1.9e-183. SS loadings = sum of squared loadings; Proportion Var = proportion variance explained; Cumulative Var = cumulative variance explained.

Table S16

Study 2 EFA factor loading matrix for the two factor solution (reduced item set).

Item	Factor 1	Factor 2
ang3	.70	
ang5	.68	
ang8	.74	
ang9_r	.60	
ang10_r	.59	
ang13_r	.55	
ang15_r	.66	
anx2	.73	
anx4	.64	
anx6	.69	
anx7	.70	
anx8	.58	
anx9_r	.56	
anx10_r	.65	
anx13_r	.69	
anx14_r	.76	
anx16_r	.57	
sad1	.63	
sad3	.56	
sad5	.67	
sad6	.68	
sad7	.68	
sad16_r	.75	
impg1		.79
impg2		.74
impg3		.82
impg6_r		.65
impg7_r		.65
impg8_r		.56
impg10_r		.65
imps1		.56
imps3		.61
impw1		.74
impw2		.72
impw3_r		.55
impw4_r		.62
