# Supplementary material for: Introduction and behavioral validation of the climate change distress and impairment scale
Source: Sci Rep. 2023 Jul 12;13:11272. doi: 10.1038/s41598-023-37573-4 (PMC10338517; doi:10.1038/s41598-023-37573-4)
Supplement: Supplementary file 17 — Supplementary Table S17. [file 41598_2023_37573_MOESM17_ESM.pdf]

Table S17

Study 2 CFA results for latent variables.

	Est	*SE*	*z*	*p*(>|z|)
Distress				
ang5	0.84	0.05	17.91	.000
ang8	0.81	0.04	19.75	.000
ang9_r	0.54	0.04	12.41	.000
ang10_r	0.43	0.04	11.55	.000
ang15_r	0.55	0.04	12.82	.000
anx2	0.69	0.04	17.70	.000
anx7	0.67	0.04	17.25	.000
anx8	0.62	0.05	13.65	.000
anx9_r	0.55	0.05	10.91	.000
anx16_r	0.44	0.04	10.33	.000
sad1	0.71	0.04	16.01	.000
sad5	0.52	0.03	15.67	.000
sad6	0.57	0.04	16.12	.000
sad7	0.59	0.04	15.56	.000
sad16_r	0.57	0.04	15.69	.000
Impairment				
impg1	0.80	0.04	20.78	.000
impg2	0.77	0.04	18.53	.000
impg3	0.79	0.04	21.85	.000
impg7_r	0.68	0.05	12.49	.000
impg8_r	0.62	0.06	10.67	.000
impg10_r	0.70	0.05	13.20	.000
imps3	0.51	0.04	12.69	.000
impw2	0.67	0.04	18.05	.000

Note. Table is continued on the next page. *Est* = Estimate, *SE* = Standard Error for *z*.

Table S17 Continued

Study 2 CFA results for latent variables.

	Est	*SE*	*z*	*p*(>|z|)
Method Factor				
ang9_r	0.24	0.05	5.29	.000
ang10_r	0.21	0.04	5.50	.000
ang15_r	0.38	0.04	8.64	.000
anx9_r	0.31	0.05	5.77	.000
anx16_r	0.24	0.05	5.23	.000
sad16_r	0.29	0.04	8.09	.000
impg7_r	0.59	0.05	12.35	.000
impg8_r	0.59	0.05	11.06	.000
impg10_r	0.44	0.05	8.89	.000

Note. *Est* = Estimate, *SE* = Standard Error for *z*.

Table S17

Study 2 CFA results for covariances.

	Est	*SE*	*z*	*p*(>|z|)
Distress				
Impairment	0.29	0.05	5.87	.000
Method Factor	0.09	0.07	1.23	.219
Impairment				
Method Factor	-0.12	0.08	-1.57	.117

Note. *Est* = Estimate, *SE* = Standard Error for *z*.

Table S17

Study 2 CFA results for variances.

	Est	*SE*	*z*	*p*(>|z|)
Distress	1.00			
Impairment	1.00			
Method Factor	1.00			
ang5	0.57	0.04	13.38	.000
ang8	0.38	0.03	12.76	.000
ang9_r	0.56	0.04	14.01	.000
ang10_r	0.42	0.03	14.04	.000
ang15_r	0.46	0.04	12.79	.000
anx2	0.40	0.03	13.44	.000
anx7	0.40	0.03	13.55	.000
anx8	0.67	0.05	14.22	.000
anx9_r	0.80	0.06	14.01	.000
anx16_r	0.59	0.04	14.17	.000
sad1	0.57	0.04	13.83	.000
sad5	0.33	0.02	13.90	.000
sad6	0.36	0.03	13.81	.000
sad7	0.43	0.03	13.92	.000
sad16_r	0.30	0.02	12.67	.000

Note. Table is continued on the next page for items assessing impairment. *Est* = Estimate, *SE* = Standard Error for *z*.

Table S17 Continued

Study 2 CFA results for variances.

	Est	*SE*	*z*	*p*(>|z|)
impg1	0.29	0.03	11.33	.000
impg2	0.41	0.03	12.62	.000
impg3	0.23	0.02	10.43	.000
impg7_r	0.41	0.04	9.74	.000
impg8_r	0.62	0.05	11.45	.000
impg10_r	0.59	0.05	12.67	.000
imps3	0.54	0.04	14.16	.000
impw2	0.34	0.03	12.82	.000

Note. *Est* = Estimate, *SE* = Standard Error for *z*.
