# Supplementary material for: Introduction and behavioral validation of the climate change distress and impairment scale
Source: Sci Rep. 2023 Jul 12;13:11272. doi: 10.1038/s41598-023-37573-4 (PMC10338517; doi:10.1038/s41598-023-37573-4)
Supplement: Supplementary file 20 — Supplementary Table S20. [file 41598_2023_37573_MOESM20_ESM.pdf]

Table S20

Study 3 CFA results for latent variables.

	Est	*SE*	*z*	*p*(>|z|)
Distress				
ang5	0.68	0.05	12.71	.000
ang8	0.68	0.04	15.56	.000
ang9_r	0.40	0.04	8.96	.000
ang10_r	0.36	0.04	8.57	.000
ang15_r	0.51	0.05	10.76	.000
anx2	0.73	0.05	16.19	.000
anx7	0.75	0.04	18.32	.000
anx8	0.62	0.05	12.47	.000
anx9_r	0.42	0.06	7.42	.000
anx16_r	0.44	0.05	9.56	.000
sad1	0.65	0.05	13.79	.000
sad5	0.65	0.04	16.02	.000
sad6	0.67	0.04	17.69	.000
sad7	0.78	0.04	19.57	.000
sad16_r	0.47	0.04	10.50	.000
Impairment				
impg1	0.77	0.05	15.76	.000
impg2	0.76	0.05	71.00	.000
impg3	0.87	0.04	19.53	.000
impg7_r	0.59	0.05	12.08	.000
impg8_r	0.46	0.05	9.30	.000
impg10_r	0.59	0.06	10.66	.000
imps3	0.75	0.04	17.52	.000
impw2	0.75	0.04	17.12	.000

Note. Tables is continued on the next page. *Est* = Estimate, *SE* = Standard Error for *z*.

Table S20 Continued

Study 3 CFA results for latent variables.

	Est	*SE*	*z*	*p*(>|z|)
Method Factor				
ang9_r	0.55	0.04	13.24	.000
ang10_r	0.48	0.04	11.98	.000
ang15_r	0.64	0.04	15.08	.000
anx9_r	0.54	0.06	9.83	.000
anx16_r	0.49	0.04	11.17	.000
sad16_r	0.56	0.04	14.06	.000
impg7_r	0.61	0.05	12.64	.000
impg8_r	0.61	0.05	12.05	.000
impg10_r	0.49	0.06	8.83	.000

Note. *Est* = Estimate, *SE* = Standard Error for *z*.

Table S20

Study 3 CFA results for covariances.

	Est	*SE*	*z*	*p*(>|z|)
Distress				
Impairment	0.19	0.06	3.48	.001
Method Factor	-0.13	0.07	-1.80	.072
Impairment				
Method Factor	-0.01	0.06	-0.11	.913

Note. *Est* = Estimate, *SE* = Standard Error for *z*.

Table S20

Study 3 CFA results for variances.

	Est	*SE*	*z*	*p*(>|z|)
Distress	1.00			
Impairment	1.00			
Method Factor	1.00			
ang5	0.77	0.06	12.98	.000
ang8	0.44	0.04	12.46	.000
ang9_r	0.37	0.03	11.79	.000
ang10_r	0.37	0.03	12.23	.000
ang15_r	0.34	0.03	10.80	.000
anx2	0.45	0.04	12.31	.000
anx7	0.31	0.03	11.59	.000
anx8	0.67	0.05	13.02	.000
anx9_r	0.78	0.06	12.79	.000
anx16_r	0.46	0.04	12.42	.000
sad1	0.56	0.04	12.82	.000
sad5	0.37	0.03	12.35	.000
sad6	0.29	0.02	11.84	.000
sad7	0.26	0.02	10.96	.000
sad16_r	0.33	0.03	11.35	.000
impg1	0.54	0.05	12.03	.000
impg2	0.41	0.04	11.48	.000
impg3	0.31	0.03	9.92	.000
impg7_r	0.51	0.05	11.27	.000
impg8_r	0.60	0.05	11.84	.000
impg10_r	0.80	0.06	12.51	.000
imps3	0.36	0.03	11.23	.000
impw2	0.38	0.03	11.43	.000

Note. *Est* = Estimate, *SE* = Standard Error for *z*.
