# Supplementary material for: Introduction and behavioral validation of the climate change distress and impairment scale
Source: Sci Rep. 2023 Jul 12;13:11272. doi: 10.1038/s41598-023-37573-4 (PMC10338517; doi:10.1038/s41598-023-37573-4)
Supplement: Supplementary file 21 — Supplementary Table S21. [file 41598_2023_37573_MOESM21_ESM.pdf]

Table S21

Study 4 list of administered items (final item set in German language).

Reverse Score	Item Type	Item
	Anger	Ich bin wütend, wenn ich sehe, wie wenig gegen den Klimawandel getan wird.
	Anxiety	Wenn ich an den Klimawandel denke, sorge ich mich um die Zukunft.
R	Sadness	Ich bin nicht traurig über den Klimawandel.
	Anger	Ich bin wütend darüber, dass wir viele Chancen verpasst haben, den Klimawandel zu stoppen.
R	Anxiety	Ich habe keine Angst um meine Zukunft auf diesem Planeten.
	Sadness	Nachrichten über den Klimawandel bedrücken mich.
R	Anger	Ich bin nicht sauer, wenn andere dem Klima schaden.
	Anxiety	Die Ungewissheit darüber, wie der Klimawandel voranschreiten wird, macht mir Angst.
	Sadness	Ich bin traurig darüber, dass Menschen und Tiere unter dem Klimawandel leiden.
R	Anger	Ich rege mich nicht auf, wenn andere den Klimawandel ignorieren.
	Anxiety	Ich habe Angst davor, dass Menschen durch den Klimawandel ihr zu Hause verlieren.
	Sadness	Ich bin traurig darüber, dass sich manche Teile der Umwelt nicht mehr von den Auswirkungen des Klimawandels erholen werden.
R	Anger	Ich bin nicht wütend darüber, dass manche Länder ihre Klimaschutzziele verfehlt haben.
	Sadness	Die Auswirkungen, die der Klimawandel auf den Planeten hat, machen mich traurig.
R	Anxiety	Ich fühle mich unbeschwert, wenn ich an den Klimawandel denke.

Note. Table is continued on the next page for items assessing impairment. Items were administered in the same order as presented here.

Table S21 Continued

Study 4 list of administered items (final item set in German language).

Reverse Score	Item Type	Item
	Impairment	Der Klimawandel raubt mir alle Energie.
R	Impairment	Meine Gedanken und Gefühle zum Klimawandel beeinflussen nicht, wie gut ich schlafe.
	Impairment	Wenn ich über den Klimawandel nachdenke, bekomme ich Kopf- oder Bauchschmerzen.
	Impairment	Wegen des Klimawandels bin ich vom Alltag überfordert.
R	Impairment	Meine Gedanken und Gefühle zum Klimawandel beeinträchtigen meinen Alltag nicht.
R	Impairment	Ich habe keine Schwierigkeiten damit, den Klimawandel gedanklich auszublenden.
	Impairment	Ständige Diskussionen über den Klimawandel beeinträchtigen meine Beziehungen.
	Impairment	Wenn ich an den Klimawandel denke, kann ich mich nicht aufraffen, zu arbeiten/lernen.

Note. Items were administered in the same order as presented here.
