# Supplementary material for: Introduction and behavioral validation of the climate change distress and impairment scale
Source: Sci Rep. 2023 Jul 12;13:11272. doi: 10.1038/s41598-023-37573-4 (PMC10338517; doi:10.1038/s41598-023-37573-4)
Supplement: Supplementary file 22 — Supplementary Table S22. [file 41598_2023_37573_MOESM22_ESM.pdf]

Table S22

Study 4 CFA results for latent variables.

	Est	*SE*	*z*	*p*(>|z|)
Distress				
dis1	1.16	0.50	23.80	0.000
dis2	1.17	0.05	23.68	0.000
dis3_r	0.53	0.06	8.49	0.000
dis4	1.12	0.50	22.88	0.000
dis5_r	0.63	0.06	11.05	0.000
dis6	1.03	0.44	23.47	0.000
dis7_r	0.48	0.06	8.32	0.000
dis8	1.05	0.05	22.83	0.000
dis9	0.92	0.04	22.42	0.000
dis10_r	0.54	0.07	8.14	0.000
dis11	0.96	0.04	21.58	0.000
dis12	0.92	0.41	22.12	0.000
dis13_r	0.50	0.06	7.77	0.000
dis14	1.08	0.04	25.72	0.000
dis15_r	0.99	0.05	21.61	0.000
Impairment				
imp1	0.70	0.04	17.60	0.000
imp2_r	1.33	0.28	4.81	0.000
imp3	0.76	0.04	17.22	0.000
imp4	0.65	0.03	20.02	0.000
imp5_r	1.72	0.41	4.15	0.000
imp6_r	1.42	0.36	3.93	0.000
imp7	0.36	0.40	9.28	0.000
imp8	0.43	0.03	13.73	0.000

Note. Table is continued on the next page. *Est* = Estimate, *SE* = Standard Error for *z*.

Table S22

Study 4 CFA results for latent variables.

	Est	*SE*	*z*	*p*(>|z|)
Method Factor				
dis3_r	0.16	0.07	2.37	0.018
dis5_r	-0.05	0.06	-.0.87	0.387
dis7_r	0.16	0.06	2.46	0.014
dis10_r	0.18	0.07	2.47	0.014
dis13_r	0.12	0.07	1.70	0.089
dis15_r	0.24	0.04	6.81	0.000
imp2_r	0.88	0.25	3.51	0.000
imp5_r	1.35	0.37	3.64	0.000
imp6_r	1.18	0.32	3.65	0.000

Note. *Est* = Estimate, *SE* = Standard Error for *z*.

Table S22

Study 4 CFA results for covariances.

	Est	*SE*	*z*	*p*(>|z|)
Distress				
Impairment	0.40	0.04	9.18	.000
Method Factor	-0.19	0.09	-2.10	.036
Impairment				
Method Factor	-0.85	0.08	-10.76	.000

Note. *Est* = Estimate, *SE* = Standard Error for *z*.

Table S22

Study 4 CFA results for variances.

	Est	*SE*	*z*	*p*(>|z|)
Distress	1.00			
Impairment	1.00			
Method Factor	1.00			
dis1	0.48	0.03	14.16	0.000
dis2	0.33	0.02	13.38	0.000
dis3_r	1.57	0.10	15.52	0.000
dis4	0.54	0.04	14.42	0.000
dis5_r	1.30	0.08	15.52	0.000
dis6	0.40	0.03	14.26	0.000
dis7_r	1.35	0.09	15.51	0.000
dis8	0.47	0.03	14.43	0.000
dis9	0.40	0.03	14.52	0.000
dis10_r	1.75	0.11	15.52	0.000
dis11	0.49	0.03	14.69	0.000
dis12	0.41	0.03	14.59	0.000
dis13_r	1.69	0.11	15.59	0.000
dis14	0.28	0.02	13.36	0.000
dis15_r	0.34	0.03	13.08	0.000
imp1	0.42	0.03	12.38	0.000
imp2_r	0.88	0.07	12.63	0.000
imp3	0.53	0.04	12.61	0.000
imp4	0.23	0.02	10.41	0.000
imp5_r	0.88	0.09	8.71	0.000
imp6_r	0.99	0.10	10.81	0.000
imp7	0.60	0.04	15.08	0.000
imp8	0.33	0.02	14.09	0.000

Note. *Est* = Estimate, *SE* = Standard Error for *z*.
